# Case scenario about TEE: Patient with dilated cardiomyopathy undergoing laparoscopic cholecystectomy

**DOI:** 10.12669/pjms.292.3077

**Published:** 2013-04

**Authors:** Peng Liang, Yuan-jing Chen, Bin Liu

**Affiliations:** 1 Peng Liang, MD, Department of Anesthesiology, West China Hospital, Sichuan University, Chengdu, China 610041.; 2 Yuan-jing Chen, MD, Department of Anesthesiology, West China Hospital, Sichuan University, Chengdu, China 610041.; 3 Bin Liu, MD, Department of Anesthesiology, West China Hospital, Sichuan University, Chengdu, China 610041.

**Keywords:** Dilated Cardiomyopathy, Pneumoperitoneum, Transesophageal Echocardiography, Central Venous Pressure

## Abstract

A 42-year-old woman, who presented with DCM (American Society of Anesthesia, ASA class IV), suffered from gallstone for years, and was scheduled for laparoscopic cholecystectomy. Echocardiography demonstrated a severely dilated left ventricle with global hypokinesia and reduction of left ventricular systolic function, ejection fraction (EF) 34% with mild mitral regurgitation and severe tricuspid regurgitation. After intubation, a transesophageal echocardiography (TEE) probe was inserted. When the IAP was gradually ascended to 14 mmHg during the laparoscopy, EF fell to 19% and the systolic pressure fell to 78 mmHg and TEE showed severely poor wall motion. But the central venous pressure (CVP) still showed about 4 mmHg throughout the whole procedure. After decreasing the IAP to 10 mmHg, we adjusted the rate of pacemaker to 70 times per minute then the systolic pressure was kept at around 100 mmHg, and the diastolic pressure was kept at 60 mmHg. EF was 30% after the reduction of IAP and the adjusting of the heart rate set. TEE is a helpful monitor in anesthesia management of patients with DCM during noncardiac surgery and CVP is useless especially for the procedure with severe hemodynamic effects.

## INTRODUCTION

Dilated cardiomyopathy (DCM) describes a group of diseases involving the myocardium. Anesthesia management of patients with DCM is challenging. As the laparoscopic (minimal invasive) operations are more and more popular and widespread, we must notice that the pneumoperitoneum may cause severe hemodynamic disorder.^1^ Management of patients with DCM undergoing laparoscopic surgery could be more complicated. With the help of transesophageal echocardiography (TEE) we may have the real-time heart function which could be of great value in guiding the anesthesia management. With the patient’s written consent for publication, we report this case about anesthesia management for patient with DCM undergoing laparoscopic cholecystectomy.

## CASE REPORT

A 42-year-old woman, who presented with DCM (American Society of Anesthesia (ASA) class IV), suffered from gallstone for years, and was scheduled for laparoscopic cholecystectomy. The symptoms of DCM were well controlled by her previous medication included diuretics, small dose of digoxin. As the preoperative electrocardiogram showed complete left bundle branch block (LBBB) and the vertigo history, the patient was assigned to receive implantation of temporary pacemaker. Preoperative echocardiography demonstrated a severely dilated left ventricle with global hypokinesia and reduction of left ventricular systolic function, ejection fraction (EF) 34% with mild mitral regurgitation and severe tricuspid regurgitation. Preoperative laboratory investigations were within the normal range except a mild increase of some myocardial enzymes.

Upon arrival into the operating room (OR), the patient’s blood pressure was 130/80 mmHg, heart rate was 80 beats/min and oxygen saturation (SpO2) was 97% on oxygen face mask 6L/min. Besides the routine monitors, a right radial arterial line was inserted after the Allen test negative, and central venous catheterization was performed to measure the central venous pressure (CVP) constantly. The rate of pacemaker was set at 60 times per minute. Defibrillator as well as lidocaine and amiodarone were prepared aside. Midazolam was given as anesthesia premedication. The patient was managed with a propofol and fentanyl-based anesthesia induction. Propofol was given through target-controlled infusion (TCI) with the effect site concentration (Ce) at 1 ug/ml at the beginning. Two separate dose of fentanyl (0.25mg) was given at the moment of induction and incision. Muscle relaxation was achieved with vecuronium. After intubation, a transesophageal echocardiography (TEE) probe was inserted. Anesthesia was maintained with sevoflurane and propofol TCI.

**Fig.1:**
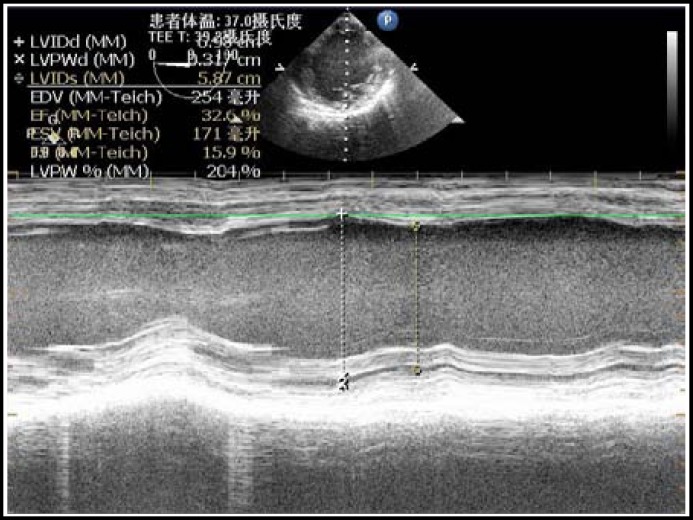
M-Mode of TEE before the surgery

**Fig.2:**
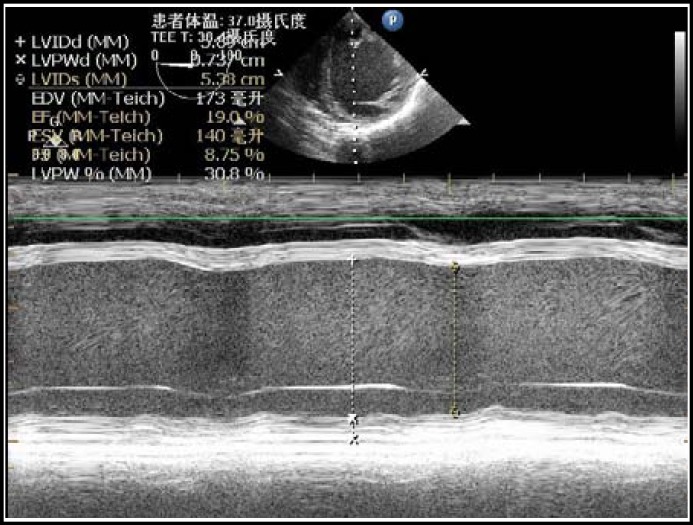
M-Mode of TEE when the intra-abdominal pressure was at 14 mmHg

The CVP was 4 mmHg and the ejection fraction (EF) showed by TEE was 32.6% (Fig.1) at the beginning. When the intra-abdominal pressure (IAP) was gradually ascended to 14 mmHg during the laparoscopy, EF fell to 19% (Fig.2) and the heart rate fell as well. 0.5mg atropine was given, but it was of no avail. The heart rate was maintained by the artificial cardiac pacemaker which was set at 60 times per minute. The systolic pressure fell to 78 mmHg. TEE still showed severely poor wall motion. After decreasing the IAP to 10 mmHg, the systolic pressure still hardly kept above 90mmHg.We adjusted the rate of pacemaker to 70 times per minute then the systolic pressure was kept at around 100 mmHg, and the diastolic pressure was kept at 60 mmHg. EF was 30% after the reduction of intra-abdominal pressure and the adjusting of the heart rate set. Continuous infusion of amrinone after a bolus dose supported the heart beat during the surgery. The surgery lasted about 20 minutes, then the patient was sent to intensive care unit without extubation. The total fluid infusion in the OR was 100 ml. The following day, the patient was discharged to the ward with stable vital signs.

## DISCUSSION

The above case represents a scenario of a patient with a typical history of DCM undergoing laparoscopic cholecystectomy. The natural history of the disease onset is difficult to determine, since asymptomatic cardiomegaly could be years before the clinical symptoms.^2^ Patients with DCM are characterized by impaired systolic function and low ejection fraction (EF<45%) with global hypokinesis of left or both ventricles. Some patients may also have right or left bundle branch block and arrhythmias such as atrial fibrillation, premature ventricular or atrial contractions and ventricular tachycardia.^3^ Based upon past experience, two key points during anesthesia management of these patients are to maintain the EF and to prevent malignant arrhythmias which may lead to sudden death.^4^

The present case had two major problems, DCM with severe cardiac dysfunction and the hemodynamic effect of laparoscopy. The principles of anesthesia for current case were maintaining her cardiac output (CO), avoiding the increase of afterload, preventing the malignant arrhythmia and alleviating the effect of pnuemoperitonieum. According to the results of TEE, we could monitor the cardiac function more directly and conveniently. In most clinical practice, the intraoperative application of TEE was limited to cardiac surgical procedures. In noncardiac surgery, the value of TEE is not determined and is limited due to its high initial cost. The familiarity in using TEE and interpretive skills of anesthesiologists does also limit the applications of perioperative TEE. However, it has been shown that TEE can influence decision making independent of the presence of CVP monitoring both in cardiac and noncardiac surgery.^5^ As for our patient, we monitored the systolic function just through the LV short-axis and four-chamber long-axis imaging, and calculated the EF under M-mode. The CVP was maintained at about 4 mmHg throughout the whole procedure, but the TEE showed a critical fall of EF and a waken wall movement of left ventricle with the increasing of IAP. Thus, instead of optimizing the preload, we should increase the CO by increasing either the stroke volume or heart rate using vasoactive or inotropic drugs.

Two major problems of laparoscopic approach are IAP increase and CO_2_ absorption from peritoneal cavity. The increased IAP can have a mechanical compression of splanchnic vascular bed, which leads to a decrease in returned blood volume and an increase in vascular resistance. At the moment of CO_2_ insufflation, stretch of peritoneum activates the vagus nerve, which results in heart rate decrease_. _The CO might not change significantly in healthy patients, but it could have a decrease in patients with cardiac disease.^6^ The hemodynamic response to pneumoperitoneum can be alleviated by limiting the degree of IAP and positioning change to head-up tilt. For this patient because of the mild mitral regurgitation and severe tricuspid regurgitation, we should keep the fast heart rate but the atropine was not valid. We did not try other drugs such as isoprenaline or epinephrine. By decreasing the IAP to 10 mmHg and adjusting the pacing heart rate to 70 beats/minute, the EF was recovered nearly preoperative level. There have been several cases about patient with DCM undergoing laparoscopic cholecystectomy (LC). Different kinds of anesthesia technique were used in those cases^7^^,^^8^, but most anesthesiologists were inclined to use epidural analgesia (EA). Aono et al compared general anesthesia (GA), epidural analgesia (EA) and GA combined with EA for LC.^9^ They suggested that EA cause mild hemodynamic changes in patients with limited cardiac function undergoing LC while anesthetics used in GA caused myocardium depression, heart rate reduction and blood vessels dilation. However, EA may exaggerate the hypotension in cardiomyopathic patient because of the vessel dilation likewise. In our case, we chose GA, the anesthetics we used in current case had minimal depressive effect on cardiac function. In addition, the anesthesia induction using TCI in our case was stable and induced less hemodynamic changes.

## CONCLUSION

In conclusion, GA facilitated by TCI provides hemodynamic stability in patients with critical cardiac condition. For these people, minimally invasive surgery is a good choice. The cardiac function was depressed severely by increasing of IAP in hemodynamic unstable patients, and the CVP may be useless compared with TEE. Meanwhile it is very important to increase the IAP very slowly in patients with critical cardiac function. TEE is a helpful monitor in anesthesia management of patients with DCM during noncardiac surgery especially for the procedure with severe hemodynamic effects.
